# LIGHT Induces Distinct Signals to Clear an AAV-Expressed Persistent Antigen in the Mouse Liver and to Induce Liver Inflammation

**DOI:** 10.1371/journal.pone.0010585

**Published:** 2010-05-14

**Authors:** Michael L. Washburn, Grigoriy I. Kovalev, Ekaterina Koroleva, Yang-Xin Fu, Lishan Su

**Affiliations:** 1 Curriculum in Genetics and Molecular Biology, University of North Carolina at Chapel Hill, Chapel Hill, North Carolina, United States of America; 2 Lineberger Comprehensive Cancer Center, University of North Carolina at Chapel Hill, Chapel Hill, North Carolina, United States of America; 3 Departments of Microbiology and Immunology, University of North Carolina at Chapel Hill, Chapel Hill, North Carolina, United States of America; 4 Department of Pathology, The University of Chicago, Chicago, Illinois, United States of America; 5 Center for Infection and Immunity, Institute of Biophysics, Chinese Academy of Sciences, Beijing, China; New York University, United States of America

## Abstract

**Background:**

Infection with adeno-associated virus (AAV) vector with liver tropism leads to persistent expression of foreign antigens in the mouse liver, with no significant liver inflammation or pathology. This provides a model to investigate antigen persistence in the liver and strategies to modulate host immunity to reduce or clear the foreign antigen expressed from AAV vector in the liver.

**Methods/Principal Findings:**

We showed that expressing LIGHT with an adenovirus vector (Ad) in mice with established AAV in the liver led to clearance of the AAV. Ad-LIGHT enhanced CD8 effector T cells in the liver, correlated with liver inflammation. LTβR-Ig proteins blocked Ad-LIGHT in clearing AAV. Interestingly, in LTβR-null mice, Ad-LIGHT still cleared AAV but caused no significant liver inflammation.

**Conclusions/Significance:**

Our data suggest that LIGHT interaction with the LTβR plays a critical role in liver inflammation but is not required for LIGHT-mediated AAV clearance. These findings will shed light on developing novel immuno-therapeutics in treating people chronically infected with hepato-tropic viruses.

## Introduction

Chronic hepatitis B virus (HBV) and hepatitis C virus (HCV) infection leads to liver diseases such as cirrhosis and hepatocellular carcinoma (HCC) [1]. Similar to HBV and HCV, adeno-associated virus (AAV) is able to establish a persistent viral infection in the liver [2]. The mechanism of these viruses to evade host T cell responses and persist in the liver is not clear. The functional impairment of T cells may contribute to persistent infection of AAV, as is observed in HBV and HCV infection in humans [3], [4].

LIGHT is a member of the TNF superfamily (TNFSF14) that interacts with the LTβR [5] and HVEM [6] receptors. Ectopic expression of LIGHT in the tumor induces a massive infiltration of T cells, correlated with expression of chemokines, adhesion molecules, and rejection of established tumors at local and distal sites [7]. Additionally, injection of an adenoviral vector expressing LIGHT (Ad-LIGHT) into tumor tissue leads to generation of tumor-specific CTL and rejection of both established and disseminated metastasizing tumor cells in the peripheral tissues of mice [8]. Stimulation of the HVEM pathway with LIGHT enhances co-stimulation of T cell activation [9]. By blocking the interaction of LIGHT with the LTβR or HVEM receptors, using a soluble HVEM-Fc or LTβR-Fc fusion protein, the allogenic T cell responses and host-specific CTL responses are significantly reduced [10], [11].

In the liver, LTβR is expressed on Kupffer cells and may be involved in the process of T cell tolerance induction [12]. Additionally, the LTβR is also expressed on hepatocytes, where it contributes to liver regeneration and liver homeostasis [13]. In transgenic mice expressing LTα-β blocking LTβR signaling reduced inflammation in the liver [14]. Since the LIGHT/LTβR-HVEM signaling pathway is involved in modulation of immune responses in the liver, we investigated their function in the liver to test whether we can induce immune activation to clear the AAV in the liver using adenovirus-mediated LIGHT expression in the liver.

## Results and Discussion

### Ad-LIGHT causes clearance of AAV genomes in the liver

**Figure 1:**
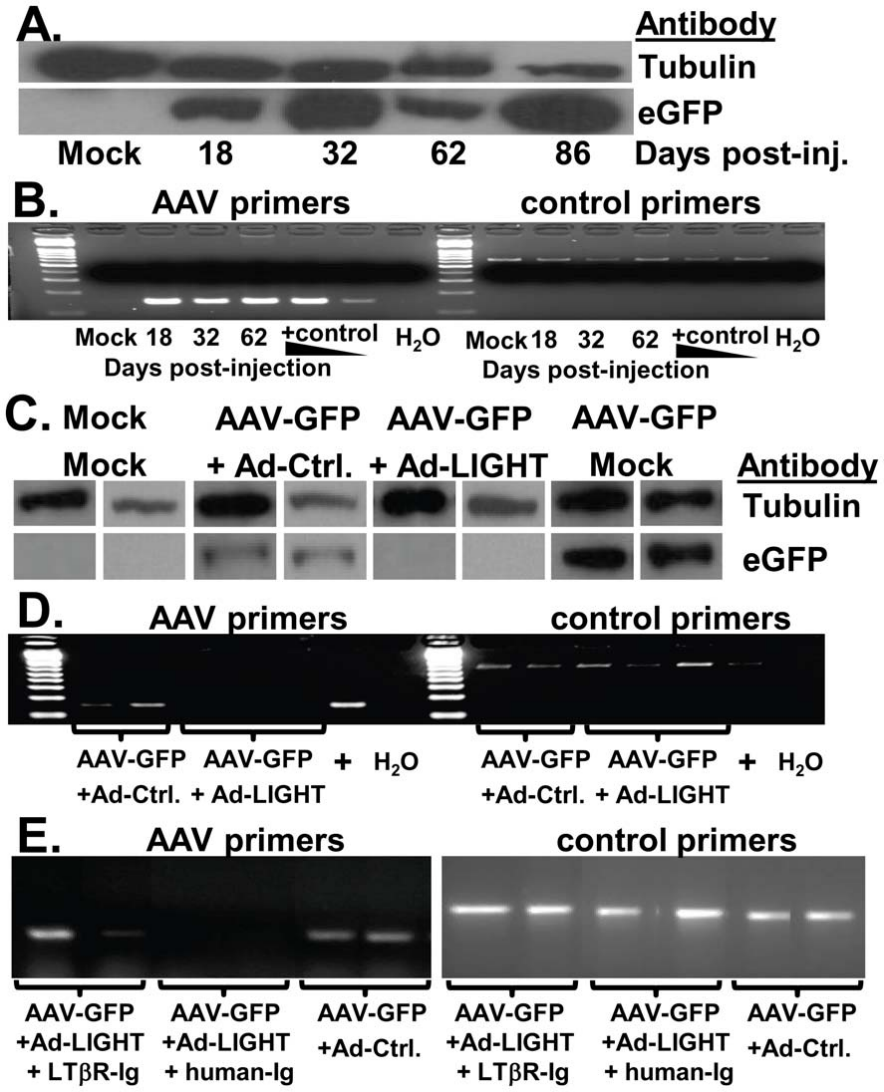
Ad-LIGHT causes clearance of AAV infection in the liver. (**A**) C57BL/6 mice were injected with PBS or AAV-GFP vector. Protein was isolated from the livers at 18 to 86 dpi. GFP protein was detected by Western blot with tubulin as a control. (**B**) Primers for GFP (AAV) were used to measure AAV genomes in the liver DNA, with p18 as a host DNA control. AAV-GFP plasmid DNA was used as a positive control. H_2_O indicates no template DNA control. (**C–E**) Mice infected with AAV-GFP for 2–3 weeks were infected with either Ad-LIGHT or Ad-Ctrl (Control) vector. Mice were sacrificed ∼4 weeks post adenovirus injection and protein or DNA was isolated from the liver. (**C**) Western blot for GFP and tubulin in the liver of two mice from a representative experiment is shown. Mock or AAV-GFP only samples were included as controls. (**D**) Liver DNA was used to detect AAV genomes after Ad-Ctrl (two mice) or Ad-LIGHT (3 mice) treatment. (**E**) Mice infected with AAV were treated with LTβR-Ig or control IgG and infected with Ad-LIGHT or Ad-Ctrl. Liver DNA was used to detect AAV genomes. Data are representative of 4 independent experiments with 2–4 mice per group in each experiment.

We used AAV packaged with serotype 8, which is liver tropic in vivo [15], and the U1a promoter was used to drive the expression of GFP in transduced hepatocytes [16]. We demonstrated that AAV infection with persistent GFP expression in the liver was efficiently established for at least 3 months. Therefore, AAV-U1a-GFP established a persistent expression of GFP in the liver with no significant liver inflammation (Fig. 1 A/B and data not shown).

The LIGHT-LTβR signaling pathway in the liver is involved in recruiting immune cells to the liver and in liver growth regulation. In addition, LIGHT is a potent co-stimulator of T cells. We postulate that the T cell tolerance induced by hepatic infection of AAV-GFP may be reversed by ectopic expression of LIGHT in the liver. To test this hypothesis, we investigated whether we can induce immune activation to clear the AAV-GFP using adenovirus-mediated LIGHT [8] expression in the liver. First, we infected C57/BL6 mice with AAV-GFP through the portal vein. After 18–21 days, mice were injected with Ad-LIGHT, Ad-Ctrl vector, or PBS. At 3–8 weeks after adenovirus injection, GFP expression and AAV genome levels in the liver were determined. Ad-LIGHT, but not Ad-Ctrl vector, diminished GFP expression in the AAV-GFP expressing liver (Fig. 1C). AAV genomes in the liver were cleared, as determined by PCR (Fig. 1D). When the LTβR-Ig fusion protein was added to the treated mice, it blocked the ability of Ad-LIGHT to clear AAV (Fig. 1E). These data indicate that ectopic expression of LIGHT in the liver can lead to clearance of an established AAV infection via interaction with LTβR.

### LIGHT-mediated clearance of AAV correlates with increased liver inflammation and CD8+ effector T cells in the liver

**Figure 2:**
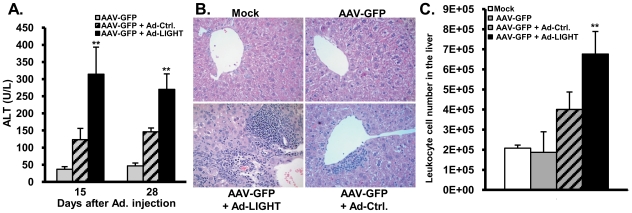
Ad-LIGHT induces liver inflammation. C57BL/6 mice were injected with PBS or AAV-GFP, and after 2–3 weeks injected with Ad-Ctrl or Ad-LIGHT. (**A**) ALT was measured from the blood at 15 and 28 days post Adenovirus injection. Error bars indicate standard deviations. (**B**) Liver sections were stained with H&E to visualize leukocyte infiltration. (**C**) Liver leukocytes were counted. Data are representative of 4 independent experiments with 3–4 mice per group in each experiment. **, p<0.01.

In LIGHT-treated mice, a significant level of liver injury was detected by ALT or intra-liver infiltration (Figure 2). Ad-Ctrl infected mice induced low levels of ALT. However, Ad-LIGHT infected mice induced a higher ALT level than Ad-Ctrl mice (Figure 2A). This is confirmed by an increase in the number of leukocytes present in the liver (Figure 2B) and by the total number of intra-hepatic leukocytes (Figure 2C). The ALT level returned to normal around 60 dpi in both Ad-LIGHT and Ad-Ctrl infected mice (data not shown).

**Figure 3:**
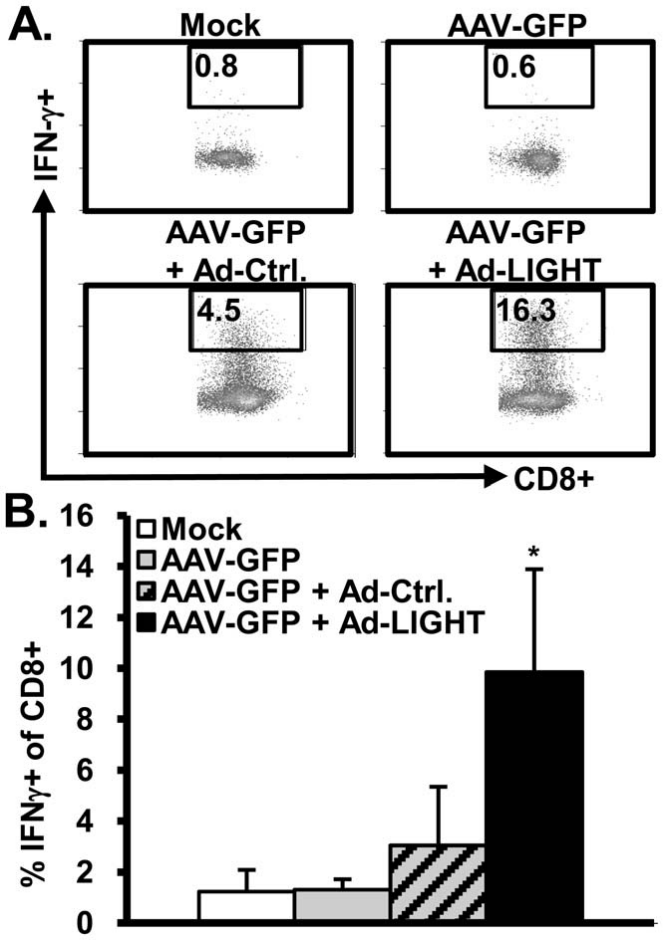
LIGHT increases CD8+ effector T cells in the liver. C57BL/6 mice previously inoculated with AAV-GFP were injected with Ad-Ctrl or Ad-LIGHT vector for 3–5 weeks. Leukocytes were isolated from the liver, and stimulated with anti-CD3 (5 ug/mL) for 18 h. The cells were then stained for surface markers and intracellular IFNγ. (**A**) A representative FACS plot of CD3+CD8+ T cells is shown for each group. Numbers are % IFNγ+ of CD8 T cells. (**B**) Summarized data are shown from 3 independent experiments with 3–4 mice per group per experiment. *, p<0.05.

To determine the immune effector cells that were stimulated by LIGHT, leukocytes were isolated from the liver and the spleen for analysis. We observed no increase in the percentage of CD4+CD44+ T cells in the liver. In contrast, there was a significant increase in the percentage of CD8+CD44+ T cells in both the liver and spleen of mice injected with Ad-LIGHT when compared to Ad-Ctrl (data not shown). We stimulated splenocytes or liver leukocytes with anti-CD3. Interestingly, there was a significant increase in the percentage of IFN-γ+ CD8+ in both the liver (Figure 3) and spleen (data not shown). LIGHT did not enhance IFN-γ expression in CD4+ cells either from the liver or spleen (data not shown). This result is consistent with reports that LIGHT is a co-stimulator of CD8+ T cells and causes an increase in IFN-γ production [9], [17], and that OT-I CD8 T cells, when transferred into mice with persistent Ova expression from an AAV vector, express IFN-γ and induce liver injury via activation of Kupffer cells [18].

### LTβR is not required for AAV clearance but is critical for LIGHT-induced liver inflammation

**Figure 4:**
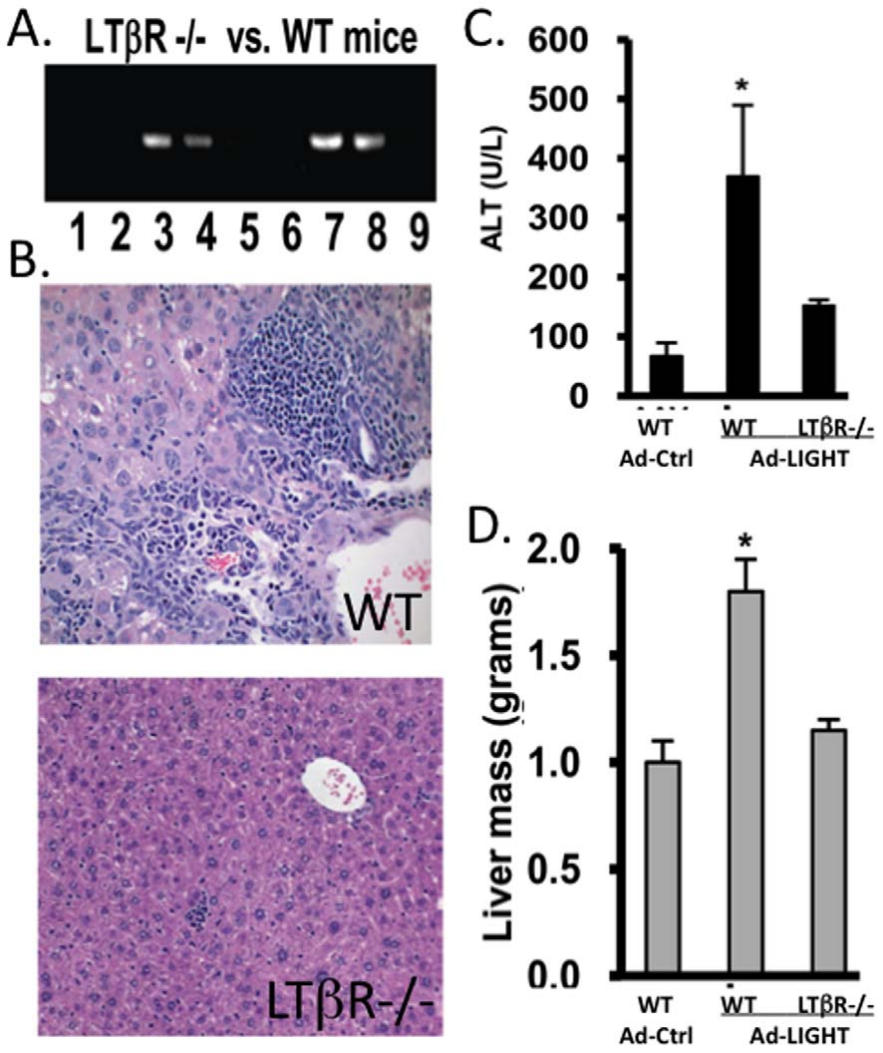
Ad-LIGHT causes no liver injury but still clears AAV in LTβR −/− mice. (**A**) Wild type or LTβR-null mice infected with AAV-GFP were injected with Ad-Ctrl or Ad-LIGHT. DNA from the liver was used to detect AAV genomes. Lanes 1&2 =  AAV−GFP + Ad-LIGHT (LTβR−/−), 3&4 =  AAV−GFP + Ad-Ctrl (LTβR−/−), 5&6 =  AAV−GFP + Ad-LIGHT (WT), 7&8 =  AAV−GFP + Ad-Ctrl (WT), lane 9 =  mock control. (**B**) Wild Type (WT) and LTβR −/− mice treated with AAV + Ad-LIGHT were sacrificed 3–4 weeks post adenovirus injection and liver sections stained with H&E. (**C**) Serum ALT was measured 21 days after Ad-LIGHT or Ad-Ctrl injection. *, p<0.05 when compared to Ad-Ctrl treatment. (**D**) The livers were weighted upon sacrifice, with an increase in liver mass in wild type but not LTβR−/− mice treated with Ad-LIGHT. The data are representative of 2 experiments with 3 mice per group per experiment.

To determine if LTβR is required for LIGHT mediated clearance of AAV and liver injury, we tested the effect of Ad-LIGHT in LTβR-null mice (Figure 4). Interestingly, Ad-LIGHT led to clearance of AAV in both wild type and mutant mice, suggesting that LTβR was not critically required for LIGHT-mediated AAV clearance (Fig. 4A). We also examined the liver injury and leukocyte infiltration. Ad-LIGHT infected wild type mice showed a higher ALT level and lymphocyte infiltration than control mice (Fig. 4B–4D). However, we observed no significant ALT induction or leukocyte infiltration into the liver of LTβR-null mice treated with Ad-LIGHT (Figure 4B–D). We conclude that the LTβR is required for LIGHT-mediated liver inflammation and injury, but a distinct signal is required for its AAV clearance activity.

In summary, delivery of Ad-LIGHT in mice with a persistent AAV infection led to clearance of AAV from the liver, correlated with elevated CD8+ effector T cells and liver injury. LTβR-Ig proteins blocked its activity. However, the LTβR was only required for LIGHT-induced liver injury but not for LIGHT-mediated AAV clearance. Therefore, LIGHT mediates distinct signals to clear AAV vectors in the liver and to induce liver inflammation. LTβR-Ig proteins may block LIGHT interaction with both LTβR and other receptors such as HVEM. The interaction of LIGHT-HVEM induces co-stimulation of CD8+ T cell activation, production of IFN-γ, and modulation of T cell responses [9], [17]. Since we observed a significant increase in effector CD8+ T cells and IFN-γ in LIGHT treated mice, the interaction of LIGHT with HVEM likely contributes to clearance of the AAV in the liver. It has been reported that LIGHT binds to LTβR or HVEM via distinct domains [9]. Our data suggest that it is feasible to generate mutant LIGHT molecules that specifically lose interaction with LTβR as novel therapeutics that can clear liver-tropic virus without liver injury.

## Materials and Methods

### Mice

Male C57BL/6 mice were purchased from the Jackson Laboratory (Bar Harbor, ME), and LTβR-null mice were maintained at the DLAM facility at the University of North Carolina at Chapel Hill. The project has been reviewed and approved by the University of North Carolina at Chapel Hill Institutional Animal Care and Use Committee (IACUC ID: 07-114.0-B, approved on 4/13/2009).

### AAV and adenovirus vectors and mouse inoculations

A self-complementary adeno-associated virus vector serotype 8 (AAV8) was kindly provided by Dr. R. Jude Samulski (Chapel Hill, NC) and used for the construction of AAV vectors expressing eGFP under the control of the U1a promoter were constructed. The recombinant Ad5 (E1/E3-) adenoviral vector expressing β-galactosidase (Ad-Ctrl) and Ad-LIGHT were generated as described [8]. To inoculate mice, 1×10^11^ vp AAV were injected through the portal vein. For Ad vector injection, 3×10^10^ vp Ad- vectors were injected intravenously 2–3 weeks after AAV inoculation.

### Antibodies

All conjugated mAb were purchased (BD Pharmingen). The JL-8 GFP antibody was from Clontech. Anti-CD3 mAB (BD Pharmingen) was used to stimulate T cells. LTβR-Ig was produced as described [19], [20]. Human control IgG was obtained from Biogen Inc. LTβR-Ig or Human IgG (200 ug/kg) was injected i.p. weekly for three weeks, once right before and twice after adenovirus injection in AAV infected mice.

### PCR, Western blotting, ALT, and liver histopathology

Serum was isolated to measure ALT levels. DNA was isolated from ∼10 mg of liver tissue using a Qiagen DNeasy kit. PCR primers of eGFP and p18 were used to quantify relative AAV genome. Protein was isolated from the liver for Western blot to detect eGFP. Liver sections were fixed in 10% formalin and paraffin sections were stained by H&E.

### Cell isolation, stimulation and flow cytometry

Liver leukocytes were isolated as described [18]. Briefly, the liver tissue suspension was treated with ACK lysis buffer to remove red blood cells. The cells were re-suspended in 40% Percoll (SIGMA) containing IMDM/10% FBS, loaded onto a 70% Percoll layer, and centrifuged for 20 minutes at 3000 rpm. Cells were stained with antibodies for CD4, CD8 and CD44. For T cell activation, 1×10^5^ spleen/LN cells were stimulated with 5 ug/ml anti-CD3 mAb for 18 h, then stained for CD4, CD8 and intracellular cytokine (IFN-γ and IL-2). Flow cytometry was performed on a CyAn FACS machine (Dako, Carpinteria, CA).
